# Evaluation of a commercial DIR platform for contour propagation in prostate cancer patients treated with IMRT/VMAT

**DOI:** 10.1002/acm2.12787

**Published:** 2020-02-14

**Authors:** Jacob E. Hammers, Sara Pirozzi, Daniel Lindsay, Orit Kaidar‐Person, Xianming Tan, Ronald C. Chen, Shiva K. Das, Panayiotis Mavroidis

**Affiliations:** ^1^ Department of Radiation Oncology University of North Carolina at Chapel Hill NC; ^2^ MIM Software Cleveland OH; ^3^ Lineberger Comprehensive Cancer Center University of North Carolina Hospitals Chapel Hill NC

**Keywords:** cone beam CT, deformable image registration, IMRT, prostate cancer, VMAT

## Abstract

**Purpose:**

To assess the performance and limitations of contour propagation with three commercial deformable image registration (DIR) algorithms using fractional scans of CT‐on‐rails (CTOR) and Cone Beam CT (CBCT) in image guided prostate therapy patients treated with IMRT/VMAT.

**Methods:**

Twenty prostate cancer patients treated with IMRT/VMAT were selected for analysis. A total of 453 fractions across those patients were analyzed. Image data were imported into MIM (MIM Software, Inc., Cleveland, OH) and three DIR algorithms (DIR Profile, normalized intensity‐based (NIB) and shadowed NIB DIR algorithms) were applied to deformably register each fraction with the planning CT. Manually drawn contours of bladder and rectum were utilized for comparison against the DIR propagated contours in each fraction. Four metrics were utilized in the evaluation of contour similarity, the Hausdorff Distance (HD), Mean Distance to Agreement (MDA), Dice Similarity Coefficient (DSC), and Jaccard indices. A subfactor analysis was performed per modality (CTOR vs. CBCT) and time (fraction). Point estimates and 95% confidence intervals were assessed via a Linear Mixed Effect model for the contour similarity metrics.

**Results:**

No statistically significant differences were observed between the DIR Profile and NIB algorithms. However, statistically significant differences were observed between the shadowed NIB and NIB algorithms for some of the DIR evaluation metrics. The Hausdorff Distance calculation showed the NIB propagated contours vs. shadowed NIB propagated contours against the manual contours were 14.82 mm vs. 8.34 mm for bladder and 15.87 mm vs. 11 mm for rectum, respectively. Similarly, the Mean Distance to Agreement calculation comparing the NIB propagated contours vs. shadowed NIB propagated contours against the manual contours were 2.43 mm vs. 0.98 mm for bladder and 2.57 mm vs. 1.00 mm for rectum, respectively. The Dice Similarity Coefficients comparing the NIB propagated contours and shadowed NIB propagated contours against the manual contours were 0.844 against 0.936 for bladder and 0.772 against 0.907 for rectum, respectively. The Jaccard indices comparing the NIB propagated contours and shadowed NIB propagated contours against the manual contours were 0.749 against 0.884 for bladder and 0.637 against 0.831 for rectum, respectively. The shadowed NIB DIR, which showed the closest agreement with the manual contours performed significantly better than the DIR Profile in all the comparisons. The OAR with the greatest agreement varied substantially across patients and image guided radiation therapy (IGRT) modality. Intra‐patient variability of contour metric evaluation was insignificant across all the DIR algorithms. Statistical significance at α = 0.05 was observed for manual vs. deformably propagated contours for bladder for all the metrics except Hausdorff Distance (*P* = 0.01 for MDA, *P* = 0.02 for DSC, *P* = 0.01 for Jaccard), whereas the corresponding values for rectum were: *P* = 0.03 for HD, *P* = 0.01 for MDA, *P* < 0.01 for DSC, *P* < 0.01 for Jaccard. The performance of the different metrics varied slightly across the fractions of each patient, which indicates that weekly contour propagation models provide a reasonable approximation of the daily contour propagation models.

**Conclusion:**

The high variance of Hausdorff Distance across all automated methods for bladder indicates widely variable agreement across fractions for all patients. Lower variance across all modalities, methods, and metrics were observed for rectum. The shadowed NIB propagated contours were substantially more similar to the manual contours than the DIR Profile or NIB contours for both the CTOR and CBCT imaging modalities. The relationship of each algorithm to similarity with manual contours is consistent across all observed metrics and organs. Screening of image guidance for substantial differences in bladder and rectal filling compared with the planning CT reference could aid in identifying fractions for which automated DIR would prove insufficient.

## 1 INTRODUCTION

Since 1983, only lung and bronchus cancer has caused more cancer deaths in men than prostate cancer.^1^ Recently, survival rates have increased due to advancements in detection and treatment methods.^2^, ^3^ Development in supporting technologies for external beam radiation therapy such as adoption of 3D‐computed tomography (CT) imaging, multileaf collimators, and advanced planning software has allowed for the clinical implementation of intensity‐modulated radiotherapy (IMRT) for the treatment of prostate cancer.^2^ The standard of care now incorporates fractional image guidance for external beam radiation therapy in the form of CT, cone beam computed tomography (CBCT), radiofrequency waves with fiducial markers, or ultrasound.^2^, ^4^ Fractional image guidance allows for precise translational corrections of patient position and alignment prior to each fractional treatment.^4^, ^5^, ^6^, ^7^, ^8^, ^9^

Adaptive radiotherapy (ART) utilizes fractional image guidance to improve conformity between the planned dose, as projected from the planning CT (pCT), and the actually delivered dose throughout the course of the treatment.^8^, ^10^ Due to the steep fall‐off of the dose distribution around the target, which is created to spare the organs‐at‐risk (OARs), a robust verification of dose delivery and quality assurance of the inverse treatment planning process is required.^4^, ^8^, ^11^, ^12^, ^13^, ^14^, ^15^, ^16^, ^17^ The high dose gradients characterizing IMRT and volumetric‐modulated arc therapy (VMAT), necessitate accurate delineation of the OARs, which are in close proximity to the target volume. This demand for accurate delineation highlights the need for consideration of inter‐fractional changes in organ position, alignment, and deformation. During treatment of prostate cancer, the degree of filling of the bladder and rectum can cause dramatic changes to size and shape of these organs throughout treatment. In order for ART to be most effective, consideration must be given to the specific anatomy for which each fraction of treatment is administered.

IMRT utilizes fractional alignment images and fiducial markers to ensure accurate localization and alignment of the patient prior to treatment. Rigid registration algorithms can provide translational and rotational corrections in patient alignment between planning CT and fractional treatments. Deformable image registration (DIR) algorithms provide superior agreement between two registered images compared to rigid registration, allowing for accurate transfer of contours of interest on one image set to another by accounting for internal organ deformations and specific fractional anatomy in a manner that rigid registration alone cannot.

Recent studies have assessed the delineation of OARs in prostate cancer for single‐modality image guidance IMRT such as CT‐on‐Rails (CTOR).^18^, ^19^, ^20^, ^21^ Contour propagation algorithm development for multi‐modality environments in the treatment of prostate cancer is less common, likely due to the challenges associated with the inferior soft tissue contrast provided in fractional CBCT image guidance. One recent study involving multiple imaging modalities, incorporated manual contour‐based approaches with DIR to generate propagated contours.^22^ In this study, the performance and limitations of a DIR algorithm, which was developed on a commercial platform were assessed on fractional CT image guided patients as well as fractional CBCT image guided patients. Furthermore, two fully automated DIR methods were compared against a shadowed contour‐based and DIR algorithm regarding their similarity to manually delineated contours. This study aims to assess the performance and limitations of contour propagation of three DIR algorithms implemented with a commercial platform for image guided prostate therapy patients treated with IMRT/VMAT.

## 2 MATERIAL AND METHODS

### 2.1 Dataset characterization

Ten prostate cancer patients treated with IMRT and 10 prostate cancer patients treated with VMAT were selected for analysis. Image guidance was applied at every fraction for all patients. For the 10 IMRT patients, the CT‐on‐rails (CTOR) system was used for image guidance, whereas the remaining 10 VMAT patients were treated using the kV‐CBCT modality of the Elekta Versa system. Each patient’s initial data included the planning CT (pCT) with contours for the targets and OARs and CTOR or CBCT scans. The image resolution of the fractional CTs was 0.98 mm × 0.98 mm with 3 mm slice thickness, while the fractional CBCTs were acquired at 1 mm × 1 mm resolution with 3 mm slice thickness. A total of 453 fractions across those 20 patients were analyzed using bladder and rectum as OARs. The patients included in this study did not receive special instructions regarding bladder filling.

### 2.2 Manual fractional contour generation

Image data were imported to MIM version 6.8 beta (MIM Software, Inc., Cleveland, OH). For 10 of the patients (five CTOR and five CBCT), the contours of bladder and rectum were manually delineated on each fraction by a single radiation oncologist. The remaining 10 patients had manually delineated contours on the initial five fractions and weekly thereafter.

### 2.3 Automated registration and contour propagation

**Figure 1:**
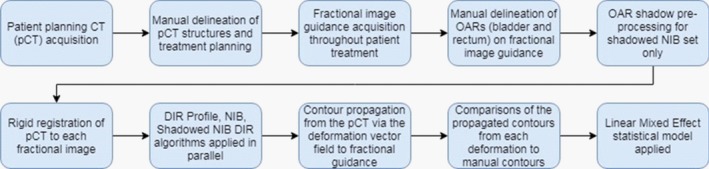
Flow chart Procedure. Graphical representation of the data processing undergone for each patient outlining the stages necessary to compare delineation methods.

**Figure 2:**
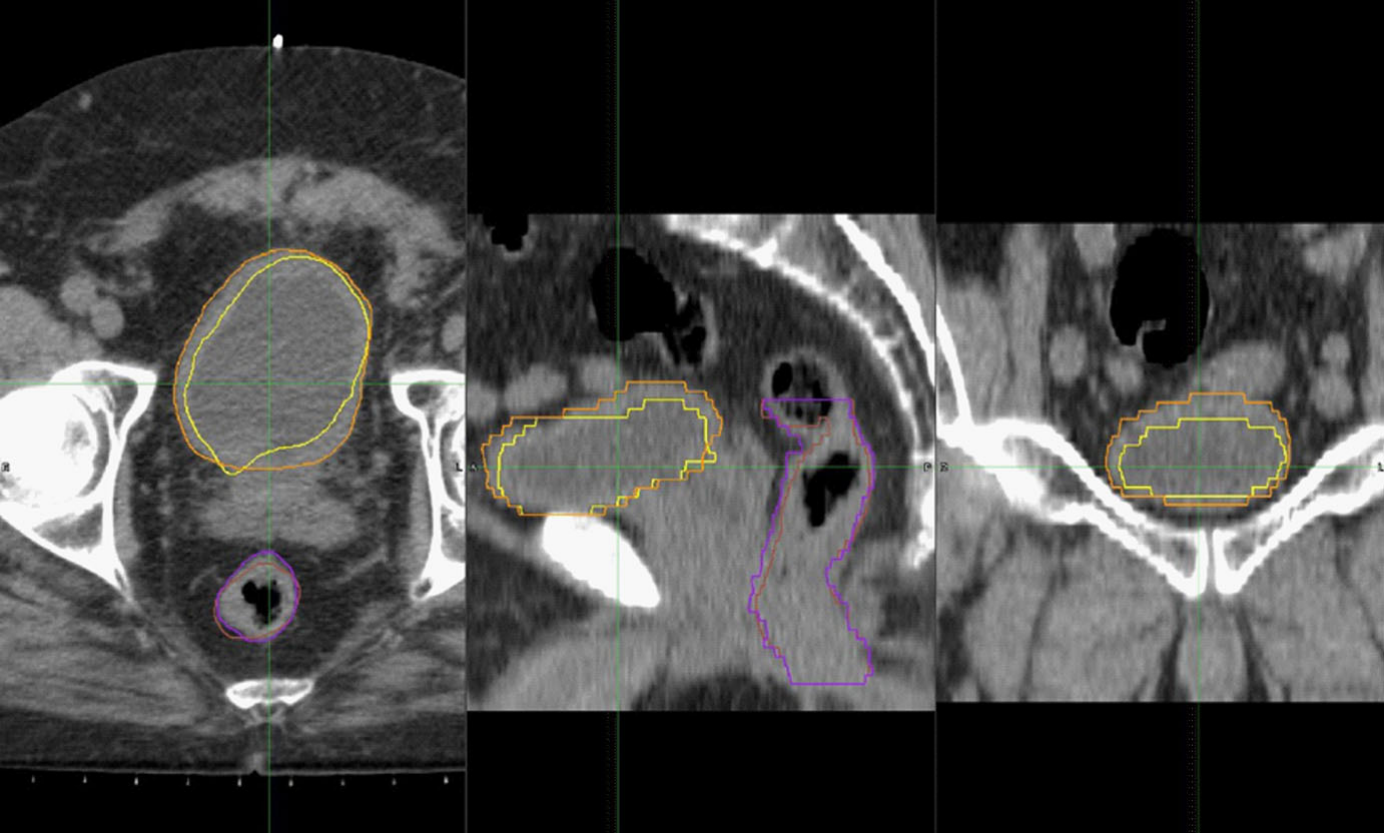
DIR Profile Propagated Contours against Manual Delineation. Fractional image guidance CT for one CTOR patient fraction with propagated contours for bladder (yellow) and rectum (brown) shown against manually delineated contours for bladder (orange) and rectum (purple) in the axial (left), sagittal (center), and coronal (right) planes.

An initial Rigid Image Registration (RIR) was applied for each fraction with the pCT as reference. A DIR processing workflow developed in collaboration with MIM Software, Inc., was implemented. The workflow employs three algorithms in parallel for the DIR process: two fully automated algorithms that are part of the MIM suite (DIR Profile algorithm and a normalized intensity‐based (NIB) DIR algorithm), and a shadowed NIB contour propagation model that was developed in conjunction with MIM (described in the next subsection). DIR Profile is a nonaffine constrained intensity‐based, free‐form deformable registration algorithm that utilizes a multi‐resolution control‐point approach when determining appropriate corresponding locations in the target volume. The NIB DIR algorithm applies normalization of intensity values to each target volume before implementing a nonaffine intensity‐based objective function defining the registration. The workflow registers each fraction for which manually delineated contours were generated with the pCT as reference. The resulting deformation vector field for each registration was applied to propagate the contours for the OARs bladder and rectum from the pCT to each daily image. A flowchart depicting a graphical representation of the data processing procedure is shown in Fig. 1. Figure 2 provides examples of typical manually drawn contours and propagated contours from the DIR Profile and NIB algorithms.

### 2.4 Shadowed NIB contour propagation

**Figure 3:**
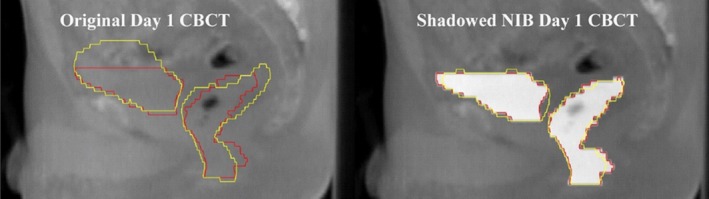
Contour Propagation Model example. Left: Original CBCT images data for the first fractional treatment of a prostate cancer patient treated with the Versa system. Contours of the bladder and rectum shown in red are the contours manually delineated in the CBCT. Contours in yellow are NIB contours propagated from the planning CT structures. Right: The same CBCT image data following contrast enhancement via the manually drown contours (red) that enables the shadowed NIB contour propagation (yellow).

This algorithm employs contour‐based approaches as well as NIB DIR. After the manual delineation of bladder and rectum on the image guided radiation therapy (IGRT) scans, a contrast enhancement of the interior regions of the defined volumes was applied through the addition of a scalar value to the Hounsfield Unit (HU) values of those regions (voxels). Rigid registration and NIB DIR were subsequently applied between each fractional scan and the respective pCT and the contours of bladder and rectum were propagated to each fractional image via the resulting deformation vector field. Figure 3 depicts a typical example of contours produced by this procedure against directly applied NIB and manually delineated contours.

### 2.5 Contour set comparison

Manual delineation and the three contour propagation DIR algorithms applied (DIR Profile, Normalized Intensity‐Based, and Shadowed Normalized Intensity‐Based) for the OARs bladder and rectum yielded six sets of comparisons for each OAR outlined below:

1. Manual vs. DIR Profile Bladder
2. Manual vs. NIB Bladder
3. Manual vs. Shadowed NIB Bladder
4. Manual vs. DIR Profile Rectum
5. Manual vs. NIB Rectum
6. Manual vs. Shadowed NIB Rectum

Four metrics for evaluation of contour similarity were utilized in the comparison of propagated contour sets against one another and the manually drawn contour sets. The metrics are defined as follows:

1. Hausdorff Distance (HD): The greatest distance of a point in one set to the closest point in another set, defined for single‐sided comparisons as

(1) $${\text{HDS}}_{\text{max}\;}\text{(A,B)}\;=\max_{a}\in_{A}\left\{d(a,\;\text{B)}\right\}$$

where A and B are sets of points, a is a single point in set A, d(a, B) denotes the minimal distance between point a and any point in set B, and HDS_max _is the single‐sided Hausdorff distance. Symmetric Hausdorff distance is calculated by:

(2) $${\text{HD}}_{\max}\left(\text{A,B}\right)=\max\left\{{\text{HDS}}_{\text{max}\;}\left(\text{A,B}\right),{\;\text{HDS}}_{\text{max}\;}\left(\text{B,A}\right)\right\}$$

A higher HD between two sets A and B indicates the existence of pockets of dissimilarity between the two sets, whereas a HD of zero indicates that the sets A and B are identical. Symmetric Hausdorff distance was utilized in all calculations and analysis for this study.

2) Mean Distance to Agreement (MDA): Mean voxelwise comparison of distance between two associative points in the contour sets A and B, defined by.

(3) $$\text{MDA}\left(\text{A,B}\right)={\text{mean}}_{a}\in_{A,\;b}\in_{B}\left\{\;\text{d(a, B) ud (b,A)}\;\right\}$$

and denotes a measure of average similarity between two contour sets. A higher MDA between two sets A and B indicates the existence of regions of dissimilarity between the two sets, where a MDA of zero indicates that the sets A and B are identical. Because MDA represents an average across all points in the sets A and B, MDA is less sensitive than HD to small pockets of high dissimilarity.

3) Dice Similarity Coefficient (DSC): Coefficient describing similarity of two regions by relating the nonoverlapping volume and the volumetric sum of the regions of interest, defined for volumes *V_1_* and *V_2_* by.

(4) $$\text{DSC}=\left(V_{1},\;V_{2}\right)=2\left|V_{1}\cap V_{2}\right|/\left|V_{1}\right|+\left|V_{2}\right|$$

where a DSC of zero indicates two volumes that do not overlap and a DSC of one indicates that the volumes *V_1_* and *V_2_* are identical.

4) Jaccard Index (JI): Ratio of the volume of overlap and the combined volume of two volumes *V_1_* and *V_2_*, defined by

(5) $$\text{JI}=\left|V_{1}\cap V_{2}\right|/\left|V_{1}\cup V_{2}\right|$$

where a JI of zero indicates two volumes that do not overlap and a JI of one indicates that the volumes *V_1_* and *V_2_* are identical.

The described metrics were calculated for each of the six comparison sets. A Linear Mixed Effect statistical model was performed for all six comparisons (Appendix). The model established 95% Confidence Intervals for each of the four metrics in every comparison and included subfactor analysis for fractional image guidance type (CTOR/CBCT) and fractional dependency.

### 2.6 Statistical analysis

We analyzed the difference of contour similarity metrics (HD, MDA, DSC, JI) between different algorithms (DIR, NIB, shadowed NIB) against manual contouring, using linear mixed effect (LME) models. Given an algorithm (e.g., DIR) and a metric (e.g., DSC), there was one observed subfactor metric value per subject per fraction of radiotherapy. The observed metric values were clustered within patients and were thus correlated. The LME model was used to account for such correlated data. Specifically, in the LME model, the metric value (e.g., DSC) was the response variable, and the fixed effect was the algorithm, DIR or manual if we compared DIR with manual, and the random effect was the subject. In addition, in the LME model, we added fraction as a covariate to adjust its effect, and we assumed the random errors over fractions (within a patient) followed an auto‐correlation structure, AR(1). The difference between an algorithm and the manual method was claimed as significant if the corresponding estimated regression coefficient was significantly different from 0, based on the Wald‐type test at a two‐sided alpha level of 0.05. The LME model analysis was conducted using SAS 9.4 (SAS Institute Cary, NC).

## 3 RESULTS

**Figure 4:**
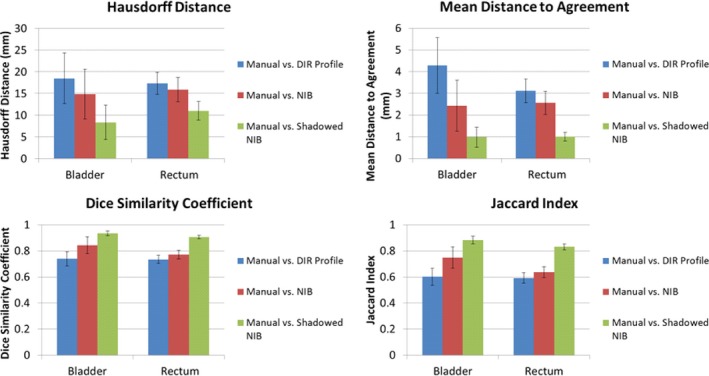
Point Estimates and Confidence Intervals for all patients (CTOR and CBCT) and metrics. Point estimates are shown as bars, where the 95% Confidence Intervals are shown as error bars for each point estimate. In each subplot, the results for the bladder (left) and rectum (right) for each contour set comparison are shown. Top left: Hausdorff Distance. Top right: Mean Distance to Agreement. Bottom left: Dice similarity Coefficient ratios. Bottom right: Jaccard Index ratios. This analysis includes all the patients (CTOR and CBCT).

**Figure 5:**
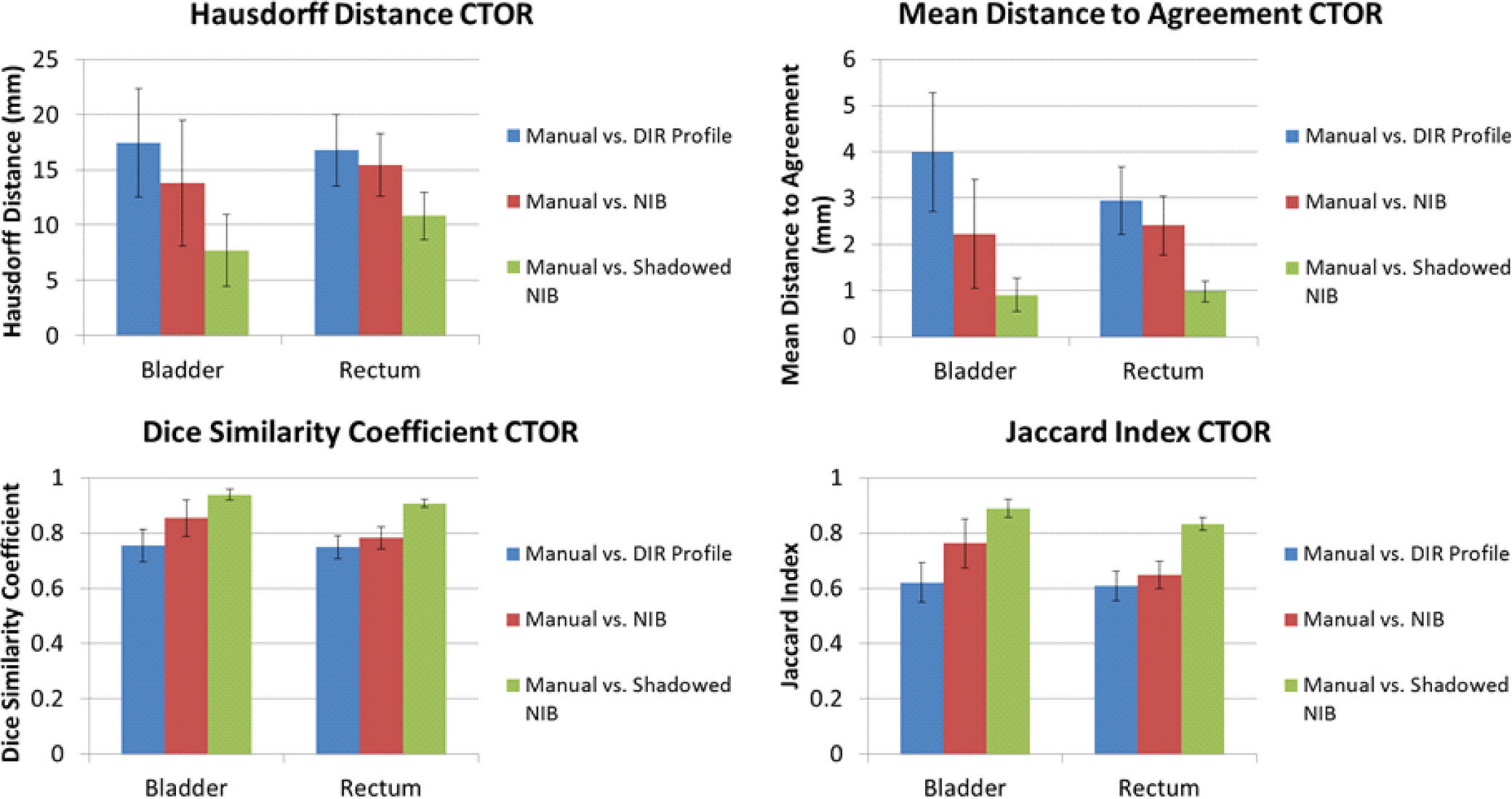
Point Estimates and Confidence Intervals for all patients (CTOR) and metrics. Point estimates are shown as bars, where the 95% Confidence Intervals are shown as error bars for each point estimate. In each subplot, the results for the bladder (left) and rectum (right) for each contour set comparison are shown. Top left: Hausdorff Distance. Top right: Mean Distance to Agreement. Bottom left: Dice similarity Coefficient ratios. Bottom right: Jaccard Index ratios. This analysis includes all the CTOR patients.

**Figure 6:**
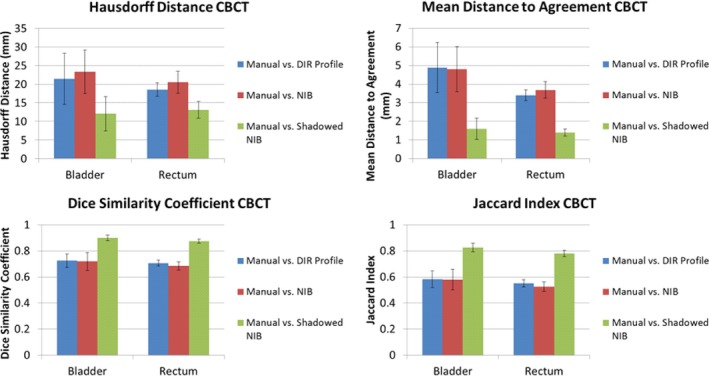
Point Estimates and Confidence Intervals for all patients (CBCT) and metrics. Point estimates are shown as bars, where the 95% Confidence Intervals are shown as error bars for each point estimate. In each subplot, the results for the bladder (left) and rectum (right) for each contour set comparison are shown. Top left: Hausdorff Distance. Top right: Mean Distance to Agreement. Bottom left: Dice similarity Coefficient ratios. Bottom right: Jaccard Index ratios. This analysis includes all the CBCT patients.

The mean values of the metrics calculated are tabulated by patient for each OAR and DIR algorithm (Appendix A). The values of the metrics show that the performance of the DIR Profile is worse than that of the NIB DIR against the manual contours (when the patients of both the CBCT and CTOR are considered together), however their differences were not statistically significant (Fig. 4). The comparison metrics of the shadowed NIB DIR method against the manual contours show significantly greater similarity than the other two contour propagation models (Fig. 4). The performance relationship of each method compared with manual contours (i.e., DIR Profile, NIB DIR, shadowed NIB DIR) is consistent across both OAR (bladder or rectum) and comparison metrics (e.g., distances). In Figs. 4, 5, 6, which describe analysis of contour propagation models compared against manual delineation, HD and MDA decrease with greater agreement between contour sets while DSC and JI increase. The shadowed NIB propagated contours were substantially more similar to the manual contours than the DIR Profile or NIB contours for both CTOR and CBCT imaging modalities (Figs. 4, 5, 6).

While the NIB propagated contours outperformed on average the DIR Profile propagated contours relative to the manual contours for the CTOR modality, the DIR Profile produced superior results for the CBCT modality compared to NIB. However their differences were not statistically significant (Figs. 5, 6). Further, the relationship of each algorithm to similarity with the manual contours is consistent across all the observed metrics and organs for each IGRT modality. While all algorithms produced superior performance for the CTOR modality compared to the CBCT modality, these differences were not statistically significant (Figs. 5, 6). For Fig. 4 and Fig. 5 (total cohort and CTOR patients only, respectively), every comparison metric indicated the same relationship of performance of the contour propagation models relative to manual delineation. For the CBCT patient cohort, Fig. 6 shows that while significantly greater agreement was shown for shadowed NIB with manual delineation than for the fully automated contour propagation models, the performance of the two fully automated contour propagation models did not statistically differ from one another, and point estimates of the metrics do not all indicate superior agreement of the same model relative to manual delineation.

Table 1 shows the average differences between the propagated contours against the manual contours for each of the evaluation metrics. With the exception of DSC and JI comparing DIR Profile propagated contours against manual delineation for the bladder, greater similarity to manual contours were observed for the CTOR cohort compared to the CBCT cohort. Table 2 displays the p‐values associated with the subfactor analysis for CBCT and fraction dependency based on the Linear Mixed Effect model analysis. In the comparisons of the subfactor metrics of NIB vs manual, and Shadowed NIB vs manual, we found significant differences (*P*‐value < 0.05) in 12 of the 16 imaging modality tests. We did not find any significant difference between DIR and manual contours in subfactor metrics. Although we controlled for fraction in the LME analysis, subfactor metrics did not vary with fraction in all comparisons, except for the comparison in MDA between Shadowed NIB vs manual for rectum (*P*‐value = 0.05).

**Table 1:** Average contour comparison metrics for the propagated contours against the manual contours per imaging modality for all patients. The unit for the metrics HD and MDA is mm.

Comparison	Modality	HD Bladder	MDA Bladder	DSC Bladder	Jaccard Bladder	HD Rectum	MDA Rectum	DSC Rectum	Jaccard Rectum
Manual vs. DIR Profile	CT	18.40	4.47	0.729	0.589	16.37	2.96	0.737	0.593
	CBCT	20.81	4.66	0.737	0.598	16.87	3.10	0.726	0.575
Manual vs. NIB	CT	15.32	2.58	0.833	0.730	15.23	2.44	0.777	0.643
	CBCT	22.57	4.68	0.713	0.573	18.49	3.29	0.710	0.557
Manual vs. Shadowed NIB	CT	7.62	0.86	0.943	0.895	9.63	0.89	0.913	0.842
	CBCT	12.24	1.51	0.906	0.833	11.25	1.31	0.879	0.785

CBCT, cone beam CT; DIR, deformable image registration; DSC, dice similarity coefficient; HD, Hausdorff distance; NIB, normalized intensity‐based; MDA, mean distance to agreement.

**Table 2:** Subfactor analysis in Linear Mixed Effect model. Every metric’s *P*‐values for the associated metrics are shown.

Comparison	Subfactor	HD Bladder	MDA Bladder	DSC Bladder	Jaccard Bladder	HD Rectum	MDA Rectum	DSC Rectum	Jaccard Rectum
Manual vs. DIR Profile	CTOR/CBCT	0.68	0.80	0.87	0.90	0.72	0.84	0.54	0.44
	Fraction	0.46	0.18	0.17	0.14	0.35	0.51	0.77	0.96
Manual vs. NIB	CTOR/CBCT	0.08	**0.01**	**0.02**	**0.01**	**0.03**	**0.01**	**<0.01**	**<0.01**
	Fraction	0.60	0.64	0.49	0.25	0.58	0.37	0.34	0.30
Manual vs. Shadowed NIB	CTOR/CBCT	0.30	0.10	**0.03**	**0.03**	0.19	**0.01**	**<0.01**	**<0.01**
	Fraction	1.00	0.48	0.32	0.38	0.06	0.05	0.18	0.19

CTOR, CT‐on‐rails; CBCT, cone beam CT; DIR, deformable image registration; DSC, dice similarity coefficient; HD, Hausdorff distance; NIB, normalized intensity‐based; MDA, mean distance to agreement.

The statistically significant values (*P*‐value < 0.05) are shown in bold.

**Figure 7:**
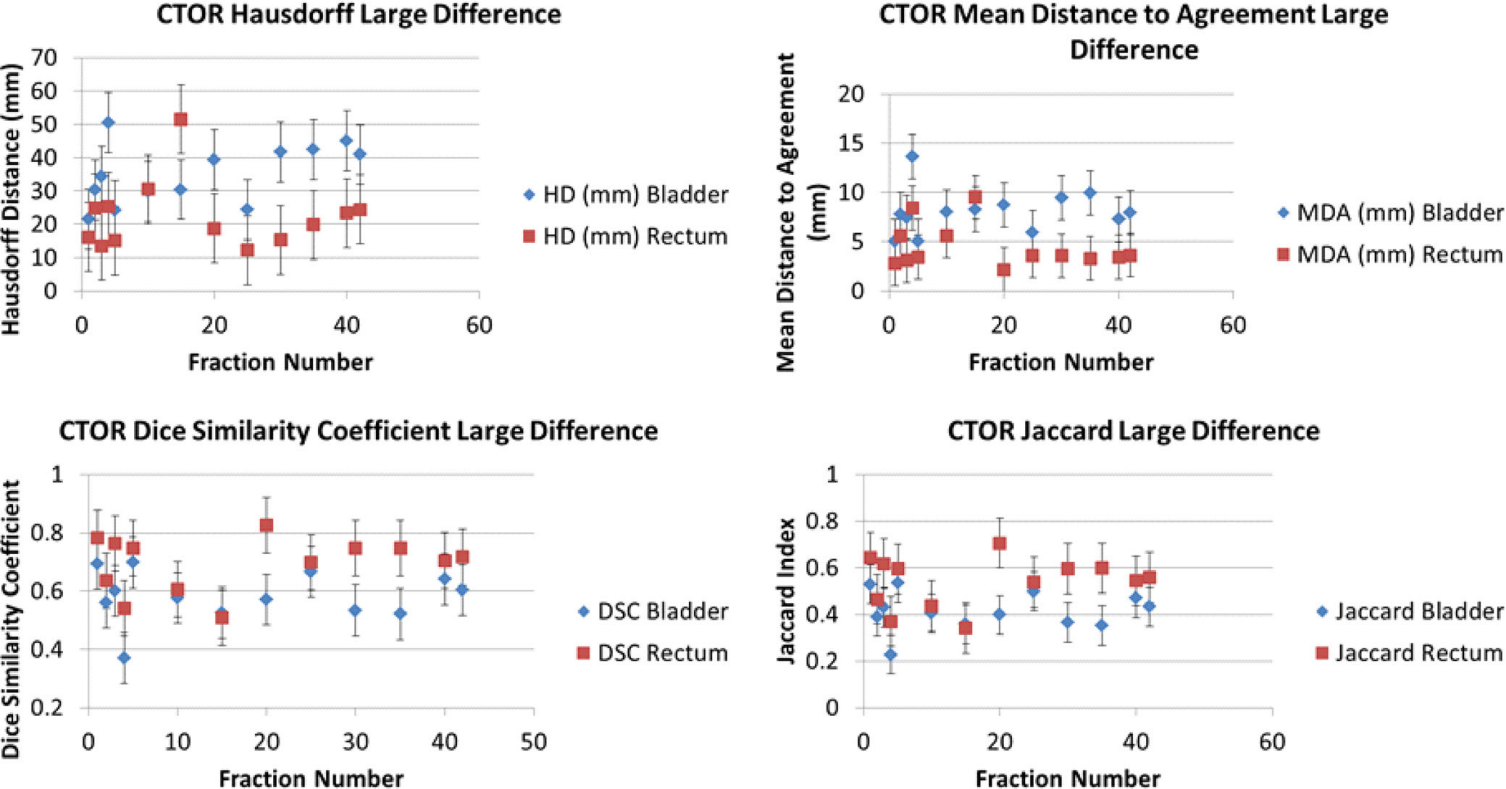
Matric evaluations for the Largest‐Difference CTOR Patient using DIR Profile Propagation. The values of the metrics are shown for each fraction and were derived by comparing the DIR Profile contour propagation with the manually delineated contours. The metrics that were used are the Hausdorff Distance (top left), Mean Distance to Agreement (top right), Dice similarity Coefficient (bottom left), and Jaccard Index (bottom right).

**Figure 8:**
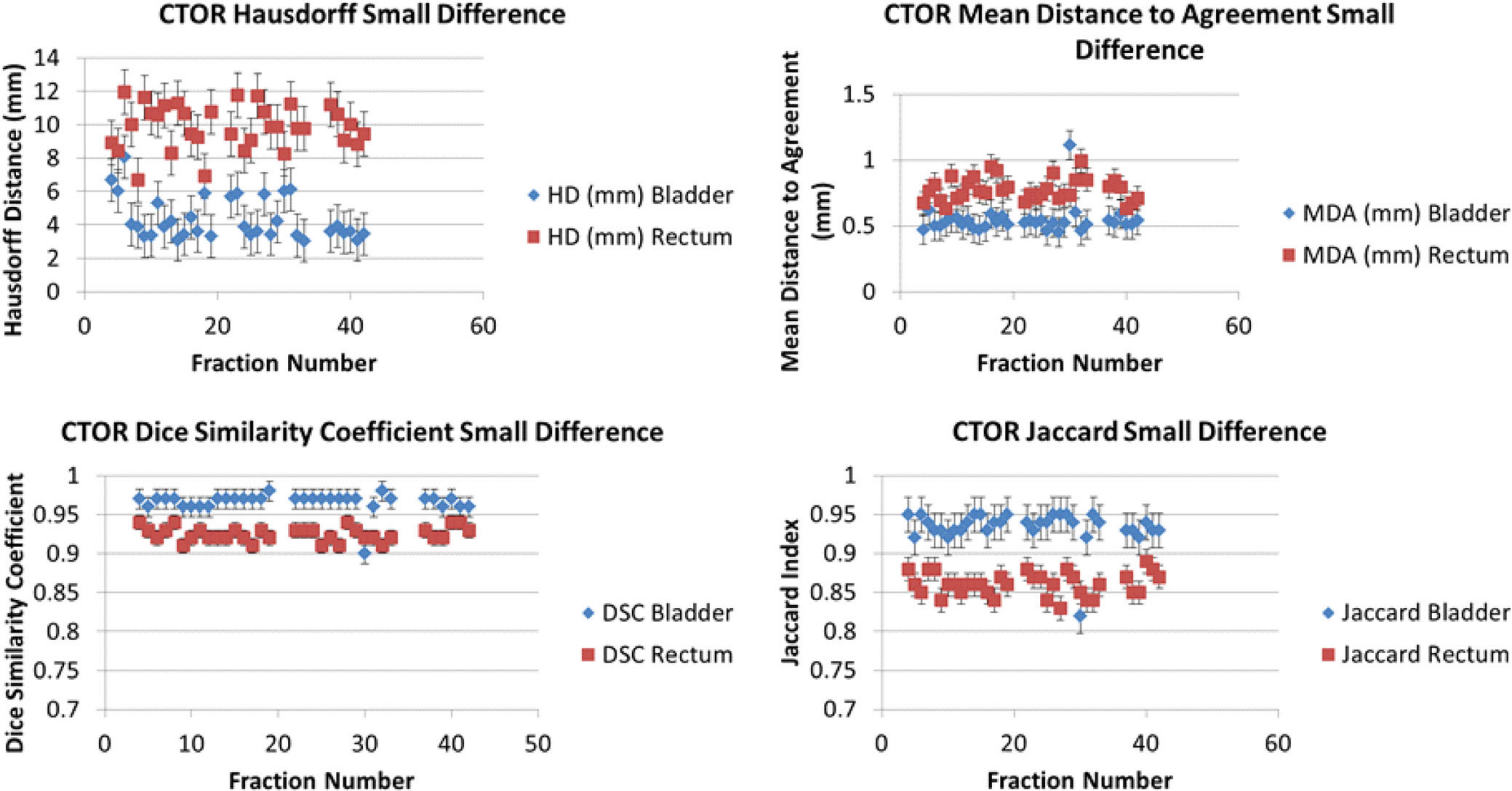
Matric evaluations for the Smallest‐Difference CTOR Patient using the shadowed NIB DIR Propagation. The values of the metrics are shown for each fraction and were derived by comparing the shadowed NIB DIR contour propagation with the manually delineated contours. The metrics that were used are the Hausdorff Distance (top left), Mean Distance to Agreement (top right), Dice similarity Coefficient (bottom left), and Jaccard Index (bottom right).

Figure 7 displays the distribution of the different metric evaluations for the CTOR patients with the largest contour disparity between delineation methods (DIR Profile). Similarly, Fig. 8 displays the metric evaluations for the CTOR patient with the smallest contour disparity between delineation methods (Shadowed NIB DIR).

**Figure 9:**
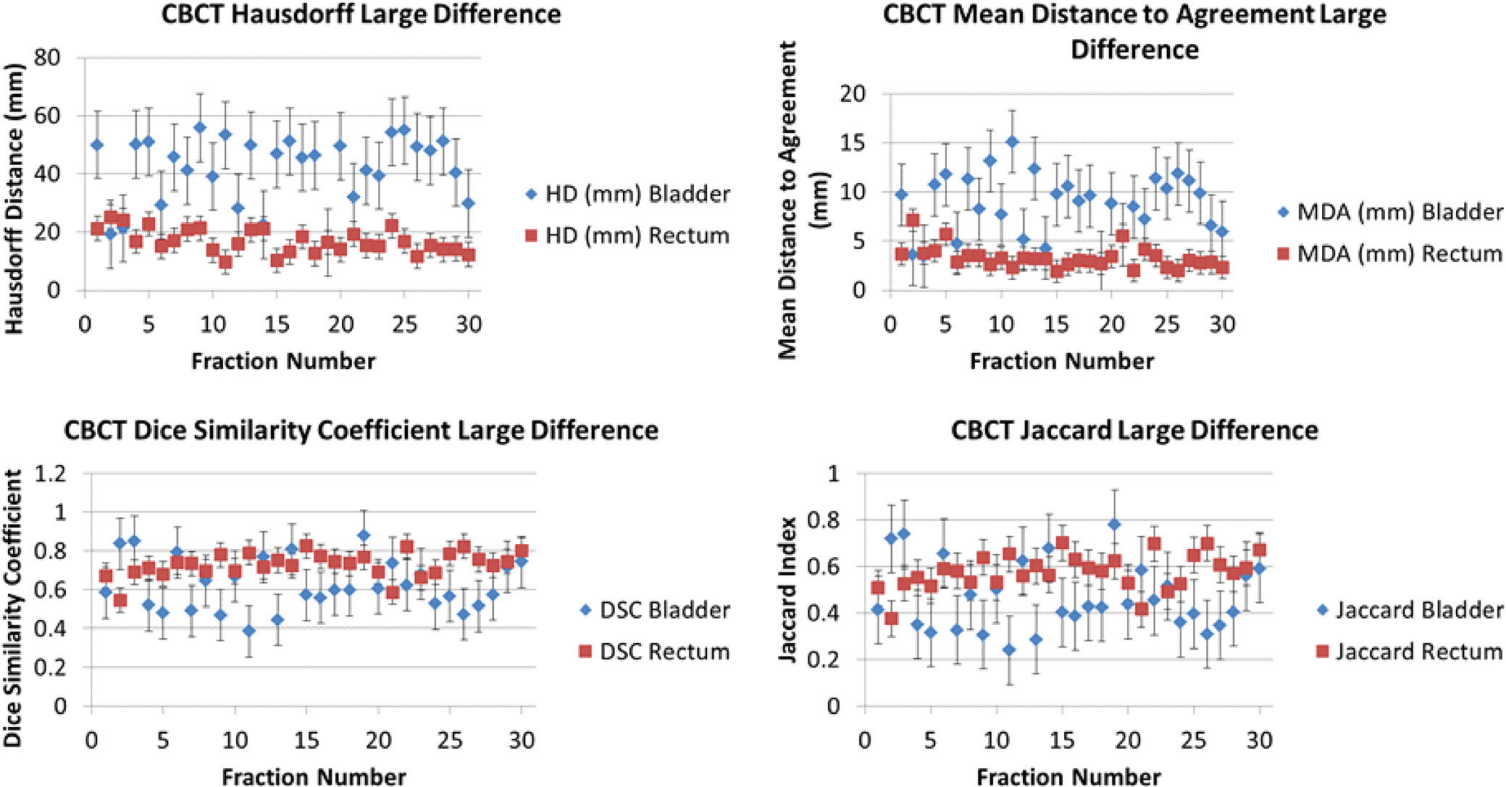
Metric evolution for the Largest‐Difference CBCT patient with DIR Profile Propagation. The values of the metrics are shown for each fraction and were derived by comparing the DIR Profile contour propagation with the manually delineated contours.

**Figure 10:**
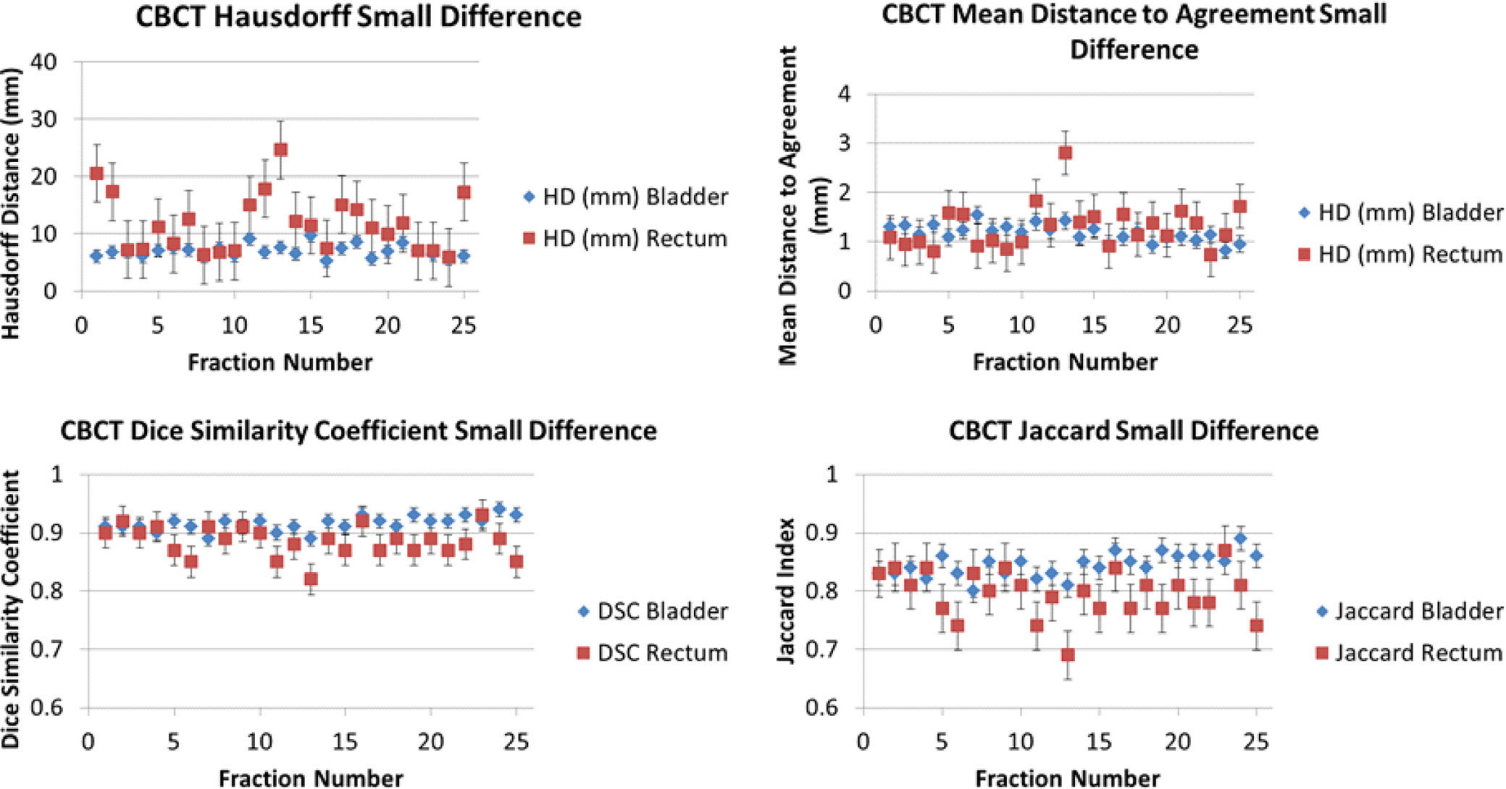
Matric evaluations for the Smallest‐Difference CBCT Patient with shadowed NIB DIR. The values of the metrics are shown for each fraction and were derived by comparing the shadowed NIB DIR contour propagation with the manually delineated contours.

Figure 9 displays the metric evaluations for the CBCT patient with the largest contour disparity between the manual and automated delineation methods (DIR Profile). Figure 10 displays the metric evaluations for the CBCT patient with the smallest contour disparity between manual and automated delineation methods. The relationship of agreement between the contour sets remains nearly constant over all the measured fractions. Nearly all the fractions for each patient possess greater agreement for the same OAR over the other, though which OAR possesses greater agreement varies substantially across patients and image guidance modality. Intra‐patient variability of contour metric evaluation was insignificant (Figs. 7, 8, 9, 10).

While the four measured contour comparison metrics are sensitive to different characteristics of similarities and dissimilarities between compared sets, a great deal of agreement among them was observed across all the patients. Because metric performance varies only slightly across fractions for a patient, weekly contour propagation models provide a reasonable approximation of fractional contour propagation models (Fig. 4).

Figure 4 depicts the 95% confidence intervals for the evaluated metrics for bladder and rectum for each contour propagation method against manually delineated contours. The Point estimates revealed the same relationship of algorithm similarity across all the metrics. The greater variability in HD and MDA against DSC and JI across all the patients indicates regions of local contour set dissimilarities, though average similarity over the entire contour set remains comparatively constant (Fig. 4). Greater contour set agreement observed for CTOR patients as opposed to CBCT patients is driven by the superior normalized‐intensity tissue contrast inherent to CTOR image guidance modality. A greater number of metrics for intensity‐based algorithms reported differences for rectum than for bladder (Table 1). This is indicative of the greater tissue contrast between bladder and surrounding tissues on CT modalities compared to rectum.

## 4 DISCUSSION

The high variance of Hausdorff distance for bladder compared to the results for the rectum indicates their difference regarding the dissimilarity of the deformed and manually contours across all fractions. This appears to result from fractions for which large deformations of the bladder caused by variable degrees of filling are not correctly captured by the DIR algorithm. The patient treatment protocol did not indicate the preferred filling state of the bladder or rectum beyond avoidance of the extreme states of empty or full. Stricter control of bladder and rectal fill status in modern treatment protocols would be likely to improve the performance of delineation algorithms in contour propagation relative to the “gold standard” of manual delineation by a physician. The large deformations that result from variable fill status of the bladder and rectum may cause the relatively high HU gradient present at the bladder wall to exceed the proximity limitation of the DIR algorithm intended to prevent excessive image warping during registration. In these cases, the resulting contour generated through DIR more closely resembles a transposition of the original bladder delineation from the reference pCT. A manual adjustment of the propagated contour is required to ensure accuracy for the fractions exhibiting sufficiently large deformations to bladder anatomy relative to the pCT. Lower variance across all modalities, methods, and metrics were observed for the rectum, most likely as a result of less volume deformation of the rectum in fractional image guidance compared with the bladder.

In this study, in order to eliminate additional sources of uncertainty (such as inter‐observer variability), the same radiation oncologists delineated the contours of bladder and rectum on all the IGRT images. Furthermore, although intra‐observer variability was not addressed or evaluated, it was not expected to be large. Usually, inter‐observer variability is larger and may have a larger impact on the accuracy of DIR due to the fact that DIR is affected significantly by image quality and extent of organ deformation. The scanning settings of both the CT‐on‐rails and the CBCT had been optimized for clinical use. However, no preprocessing steps (such as resampling volumes to isotropic spacing, CBCT artifact reduction) were applied. In the DIR Profile and NIB processes the contours of bladder and rectum were automatically created as part of the DIR workflow However, as it was shown in this study, the accuracy of this operation heavily relies on image quality, which (especially in the case of CBCT) is not adequate.

While the results of the comparisons against the manually delineated contours were consistent across bladder and rectum, the performance characteristics of the DIR algorithm methods varied. DIR Profile comparison metrics were similar to those of NIB DIR for the rectum, but three of the four calculated metrics indicated substantially improved performance of the NIB DIR for the bladder over the DIR Profile. Such improvement may be derived from the improved applicability of NIB DIR in multi‐modality in addition to single‐modality environments. While the shadowed NIB DIR incorporates a manual component that was expected to produce the significantly improved performance characteristics, which it exhibited over the fully automated methods, the comparison against a pure contour‐based approach on a large database would provide further insight. Contour‐based approaches may prove more similar to shadowed NIB DIR in performance against manual delineation, but shadowed NIB DIR is expected to provide useful characterization of the spatial information in the image that pure contour‐based approaches are unable to.

The Linear Mixed Effect statistical model applied to the generated comparison metrics was selected in collaboration with a biostatistician in order to most accurately establish estimation of the parameters while accounting for the variability introduced by patients, image guidance modality, time/fractional dependency, and manual contour delineation. The standard errors calculated in the model represent the combined effect of variability from these sources on the measured metrics for comparison among the automated propagation models and the manual contours they were designed to estimate. The expected modality dependency was identified in many of the metrics derived using the NIB DIR algorithms, while no observed modality dependency was identified for the DIR Profile contours. The lack of modality dependency for the DIR Profile results suggests that the internal restrictions of the algorithm place stricter limits on the amount of deformation allowed in the result.

In this study, the already installed of DIR algorithms of MIM were used. A data preprocessing step was involved only in the shadowed NIB case where the density of the voxels inside bladder and rectum was escalated to the density levels of bone. So, in this sense the results presented in this study can be replicated by other MIM users.

The automated contour propagation methods analyzed in this study were compared against manually delineated contours for each fraction. While the contour review by an expert physician may reduce intra‐observer variance, the substantial volume of fractions delineated prevented correction for inter‐observer variance. Further analysis against consensus‐defined contours will be possible as a result of the reduced volume associated with analysis on partial datasets that represent a reasonable approximation of all patient fractions due to the relatively small observed intra‐patient metric variance. Screening of image guidance for substantial differences in bladder and rectal filling compared with the planning CT reference could aid in identifying fractions for which automated DIR would prove insufficient.

## 5 CONCLUSION

This study provides an analysis of the reliability and consistency of automated and partially automated contour propagation methods and aims at indicating the situations in which those methods are insufficient. The shadowed NIB propagated contours were substantially more similar to the manual contours than the DIR Profile or NIB contours for both the CTOR and CBCT imaging modalities. While manual delineation of the OARs on each fractional image guidance in order to improve contour propagation is clinically infeasible, weekly fractional delineation of contours may be sufficient to establish a representation of bladder and rectal anatomy that can inform the accumulation of dose within the bladder and rectum during that period of treatment. The relationship of each algorithm to similarity with the manual contours is consistent across all observed metrics and organs. Predicting fractions for which DIR algorithms are unable to account for the changes relative to the planning CT could indicate the situations where manual delineation is necessary. By reducing the total volume of manual delineation to those fractions for which it is required to ensure accuracy, the workload required for adaptive radiotherapy and correction of treatment for internal fractional deformations can be substantially reduced. While the current “gold standard” approach of manual delineation is essential for treatment planning, pursuit of an accurate fully automated contour propagation algorithm for fractional image guidance is critical to establishing a process that is robust to the uncertainties introduced due to inter‐observer and intra‐observer variability in contour delineation for large volumes of fractions.

## CONFLICT OF INTEREST

The authors report no conflict of interest in conducting this research.
