# Supplementary material for: Evaluation of a commercial DIR platform for contour propagation in prostate cancer patients treated with IMRT/VMAT
Source: J Appl Clin Med Phys. 2020 Feb 14;21(2):14–25. doi: 10.1002/acm2.12787 (PMC7020979; doi:10.1002/acm2.12787)
Supplement: Supplementary file 1 [file ACM2-21-14-s001.pdf]

Appendix A. Statistical model and patient parameter tables

Linear Mixed Effect Model

The Linear Mixed Effect (LME) model represents an extended linear regression model for which subgroups exist within the data. Given an algorithm (e.g., DIR) and a metric (e.g., HD), there was one observed subfactor metric value per subject per fraction (of radiotherapy). The observed metric values were clustered within subjects (patients) and were thus correlated for a metric y_{ij} denoting a parameter observation for patient i on treatment j via the following equation:

$$Y_{ij} = a_i + a_0 + a_1*M + a_2*j + e_{ij} ,$$

where
Y_{ij} = The LME model parameter
a_i = The patient-specific random effect
a_0 = The group mean parameter of interest (e.g., mean Hausdorff distance)
a_1*M = The patient-specific fixed effect for modality parameter
a_2*j = The time (treatment order) sequence of fraction observations, accounting for possible time trend
e_{ij} = The random error term.

Table 1. Average metric evaluations for CTOR patients comparing manual contours against DIR Profile propagated contours.

CTOR Manual vs. DIR Profile	HDBladder	MDABladder	DSCBladder	JaccardBladder	HDRectum	MDARectum	DSCRectum	JaccardRectum
CTOR1	25.71	7.37	0.62	0.47	8.53	1.09	0.87	0.77
CTOR2	23.32	5.01	0.71	0.55	19.81	3.73	0.66	0.50
CTOR3	16.33	4.45	0.72	0.57	11.76	2.04	0.76	0.62
CTOR4	17.35	4.42	0.71	0.56	19.70	3.51	0.73	0.59
CTOR5	16.07	4.36	0.79	0.66	16.75	2.49	0.75	0.61
CTOR6	17.20	3.58	0.81	0.68	18.43	4.09	0.69	0.54
CTOR7	34.99	8.02	0.58	0.41	22.35	4.46	0.69	0.54
CTOR8	17.85	3.35	0.69	0.53	22.36	4.35	0.68	0.52
CTOR9	9.24	1.87	0.82	0.70	15.10	2.64	0.75	0.60
CTOR10	10.67	2.65	0.84	0.73	13.33	2.29	0.78	0.64
**Mean**	**18.87**	**4.51**	**0.73**	**0.59**	**16.81**	**3.07**	**0.74**	**0.59**
**StdDev**	7.12	1.83	0.08	0.10	4.36	1.07	0.06	0.07

Table 2. Average metric evaluations for CTOR patients comparing manual contours against NIB propagated contours.

CTOR Manual vs. NIB	HDBladder	MDABladder	DSCBladder	JaccardBladder	HDRectum	MDARectum	DSCRectum	JaccardRectum
CTOR1	14.90	2.57	0.84	0.74	8.19	1.11	0.85	0.74
CTOR2	24.04	4.38	0.73	0.58	18.04	3.24	0.71	0.56
CTOR3	13.78	2.44	0.84	0.73	10.81	1.35	0.84	0.72
CTOR4	14.81	2.40	0.83	0.72	18.21	3.35	0.72	0.57
CTOR5	6.45	0.95	0.95	0.91	14.21	1.75	0.82	0.69
CTOR6	11.91	1.41	0.92	0.85	16.61	3.64	0.71	0.57
CTOR7	35.71	6.85	0.62	0.46	16.96	2.58	0.82	0.69
CTOR8	14.82	1.88	0.83	0.71	22.27	3.58	0.73	0.59
CTOR9	7.82	1.11	0.90	0.81	16.96	2.56	0.76	0.62
CTOR10	6.96	0.91	0.94	0.89	13.77	1.79	0.82	0.70
**Mean**	**15.12**	**2.49**	**0.84**	**0.74**	**15.60**	**2.49**	**0.78**	**0.65**
**StdDev**	8.43	1.76	0.10	0.13	3.81	0.90	0.05	0.07

Table 3. Average metric evaluations for CTOR patients comparing manual contours against shadowed NIB propagated contours.

CTOR Manual vs. Shadowed NIB	HDBladder	MDABladder	DSCBladder	JaccardBladder	HDRectum	MDARectum	DSCRectum	JaccardRectum
CTOR1	9.62	1.17	0.92	0.86	5.48	0.50	0.94	0.88
CTOR2	9.53	0.93	0.95	0.90	9.55	1.07	0.90	0.83
CTOR3	5.87	0.71	0.95	0.91	8.16	0.73	0.91	0.84
CTOR4	6.20	0.75	0.94	0.89	8.61	0.75	0.93	0.86
CTOR5	4.42	0.51	0.97	0.95	9.37	0.79	0.91	0.84
CTOR6	5.33	0.63	0.96	0.93	11.64	1.01	0.90	0.82
CTOR7	21.06	2.34	0.88	0.80	14.15	1.20	0.91	0.84
CTOR8	12.16	1.16	0.90	0.82	17.41	1.77	0.86	0.75
CTOR9	5.27	0.65	0.94	0.89	10.00	0.94	0.90	0.83
CTOR10	4.35	0.54	0.97	0.93	9.87	0.78	0.92	0.86
**Mean**	**8.38**	**0.94**	**0.94**	**0.89**	**10.42**	**0.95**	**0.91**	**0.84**
**StdDev**	4.90	0.52	0.03	0.05	3.15	0.33	0.02	0.03

Table 4. Average metric evaluations for CBCT patients comparing manual contours against DIR Profile propagated contours.

CBCT Manual vs. DIR Profile	HDBladder	MDABladder	DSCBladder	JaccardBladder	HDRectum	MDARectum	DSCRectum	JaccardRectum
CBCT1	42.87	9.24	0.60	0.44	17.25	3.63	0.70	0.54
CBCT2	16.73	3.90	0.68	0.52	16.29	3.13	0.72	0.56
CBCT3	19.04	3.89	0.82	0.71	12.92	2.45	0.75	0.60
CBCT4	18.52	4.37	0.72	0.58	16.86	3.28	0.73	0.58
CBCT5	10.83	2.53	0.83	0.71	18.33	2.91	0.75	0.60
CBCT6	17.24	5.34	0.68	0.52	18.33	3.08	0.70	0.54
CBCT7	11.14	2.88	0.80	0.67	21.48	3.10	0.75	0.60
CBCT8	19.73	5.10	0.71	0.57	18.13	3.25	0.70	0.54
CBCT9	38.95	7.95	0.64	0.48	21.21	4.04	0.64	0.48
CBCT10	15.64	3.65	0.77	0.63	16.31	3.50	0.73	0.58
**Mean**	**21.07**	**4.88**	**0.73**	**0.58**	**17.71**	**3.24**	**0.72**	**0.56**
**StdDev**	10.36	2.05	0.07	0.09	2.35	0.41	0.03	0.04

Table 5. Average metric evaluations for CBCT patients comparing manual contours against NIB propagated contours.

CBCT Manual vs. NIB	HDBladder	MDABladder	DSCBladder	JaccardBladder	HDRectum	MDARectum	DSCRectum	JaccardRectum
CBCT1	37.63	7.56	0.66	0.50	17.46	3.14	0.74	0.59
CBCT2	21.89	5.47	0.52	0.36	18.31	3.53	0.68	0.53
CBCT3	17.51	3.31	0.83	0.71	14.25	2.49	0.74	0.60
CBCT4	23.58	4.93	0.67	0.52	16.19	2.87	0.76	0.61
CBCT5	14.26	2.42	0.84	0.73	22.92	4.18	0.64	0.47
CBCT6	14.55	2.89	0.81	0.68	16.36	2.82	0.72	0.57
CBCT7	12.80	3.49	0.78	0.63	25.82	3.90	0.68	0.52
CBCT8	21.30	4.22	0.78	0.66	22.90	4.13	0.64	0.47
CBCT9	32.23	6.15	0.70	0.54	24.43	3.44	0.71	0.56
CBCT10	26.36	5.45	0.70	0.54	20.10	4.13	0.68	0.52
**Mean**	**22.21**	**4.59**	**0.73**	**0.59**	**19.87**	**3.46**	**0.70**	**0.55**
**StdDev**	7.69	1.53	0.09	0.11	3.75	0.58	0.04	0.05

Table 6. Average metric evaluations for CBCT patients comparing manual contours against shadowed NIB propagated contours.

CBCT Manual vs. Shadowed NIB	HDBladder	MDABladder	DSCBladder	JaccardBladder	HDRectum	MDARectum	DSCRectum	JaccardRectum
CBCT1	26.10	3.23	0.85	0.74	11.62	1.23	0.89	0.80
CBCT2	9.17	1.25	0.89	0.80	10.59	1.71	0.85	0.73
CBCT3	10.56	1.05	0.94	0.89	6.66	0.88	0.90	0.82
CBCT4	13.14	1.27	0.92	0.85	10.20	1.27	0.88	0.79
CBCT5	6.92	1.17	0.92	0.84	11.65	1.29	0.89	0.80
CBCT6	5.28	0.88	0.93	0.87	13.99	1.73	0.83	0.71
CBCT7	6.27	0.97	0.93	0.87	14.29	1.23	0.90	0.81
CBCT8	5.48	0.77	0.94	0.89	13.69	1.24	0.87	0.78
CBCT9	22.48	2.94	0.87	0.78	18.82	1.47	0.88	0.79
CBCT10	9.09	1.32	0.90	0.83	15.36	1.43	0.88	0.78
**Mean**	**11.45**	**1.48**	**0.91**	**0.84**	**12.69**	**1.35**	**0.88**	**0.78**
**StdDev**	6.87	0.82	0.03	0.05	3.15	0.24	0.02	0.03
